# Nutritional influences on Alzheimer’s disease: Insights from transformer models and explainable AI

**DOI:** 10.1002/alz.089077

**Published:** 2025-01-09

**Authors:** Ziming Liu, Longjian Liu, Robert Heidel, Xiaopeng Zhao

**Affiliations:** ^1^ University of Tennessee, Knoxville, TN USA; ^2^ Drexel University Dornsife School of Public Health, Philadelphia, PA USA; ^3^ University of Tennesse Meical Center, Knoxville, TN USA

## Abstract

**Background:**

This study utilizes transformer‐based machine learning models and explainable AI (XAI) techniques to investigate the complex relationship between various nutritional factors and AD mortality. Drawing data from the Third National Health and Nutrition Examination Survey (NHANES III 1988 to 1994) and the NHANES III Mortality‐Linked File (2019), it aims to dissect the multifaceted interactions between nutrition and AD.

**Method:**

The study employs advanced transformer models alongside traditional machine learning methods like random forests and support vector machines. These models are applied to the NHANES III dataset to predict mortality outcomes in AD, HD, and CA, with a particular focus on nutritional factors. XAI techniques, including the Shapley Additive Explanations (SHAP) and integrated gradients, are used to enhance the interpretability of these models. The aim is to identify and understand the most influential nutritional elements contributing to AD mortality and how these compare across different disease contexts.

**Result:**

Transformer models demonstrated a high degree of accuracy and reliability in identifying AD cases, outperforming traditional models in recall scores. XAI analysis highlighted several key nutritional factors significantly associated with AD mortality, such as serum apolipoprotein AI, vitamin B12, and selenium levels. These findings underscore the potential of transformer models and XAI in extracting deeper insights from complex medical data. Additionally, the study revealed distinct nutritional patterns associated with AD compared to HD and CA, suggesting specific nutritional pathways influencing the progression and mortality of these diseases.

**Conclusion:**

This research represents a significant step forward in applying advanced AI methodologies to medical research, particularly in the context of AD. By leveraging transformer models and XAI, the study provides a nuanced understanding of the impact of nutrition on AD mortality. It highlights the potential of these techniques in unraveling the complex interplay between diet and disease progression, offering a foundation for future investigations into targeted nutritional interventions for AD management. The findings also suggest broader implications for the use of AI in healthcare, paving the way for more sophisticated and personalized approaches to disease prevention and management.

